# Rare earth-based quaternary Heusler compounds *M*CoV*Z* (*M* = Lu, Y; *Z* = Si, Ge) with tunable band characteristics for potential spintronic applications

**DOI:** 10.1107/S2052252517013264

**Published:** 2017-10-06

**Authors:** Xiaotian Wang, Zhenxiang Cheng, Guodong Liu, Xuefang Dai, Rabah Khenata, Liying Wang, Abdelmadjid Bouhemadou

**Affiliations:** a School of Physical Science and Technology, Southwest University, Chongqing 400715, People’s Republic of China; b Institute for Superconducting and Electronic Materials (ISEM), University of Wollongong, Wollongong 2500, Australia; c School of Material Sciences and Engineering, Hebei University of Technology, Tianjin 300130, People’s Republic of China; d Laboratoire de Physique Quantique, de la Matière et de la Modélisation Mathématique (LPQ3M), Université de Mascara, Mascara 29000, Algeria; e Laboratory for Developing New Materials and their Characterization, University of Setif 1, Setif 19000, Algeria

**Keywords:** spin-filter materials, spin-gapless semiconductors, band structures, magnetic properties, first-principles predictions, density functional theory, materials modelling

## Abstract

Magnetic Heusler compounds (MHCs) have recently attracted great attention since these types of material provide novel functionalities in spintronic and magneto-electronic devices. Among the MHCs, some compounds have been predicted to be spin-filter semiconductors [also called magnetic semiconductors (MSs)], spin-gapless semiconductors (SGSs) or half-metals (HMs). In this work, by means of first-principles calculations, it is demonstrated that rare earth-based equiatomic quaternary Heusler (EQH) compounds with the formula *M*CoV*Z* (*M* = Lu, Y; *Z* = Si, Ge) are new spin-filter semiconductors with total magnetic moments of 3 µ_B_. Furthermore, under uniform strain, there are physical transitions from spin-filter semiconductor (MS) → SGS → HM for EQH compounds with the formula LuCoV*Z*, and from HM → SGS → MS → SGS → HM for EQH compounds with the formula YCoV*Z*. Remarkably, for YCoV*Z* EQH compounds there are not only diverse physical transitions, but also different types of spin-gapless feature that can be observed with changing lattice constants. The structural stability of these four EQH compounds is also examined from the points of view of formation energy, cohesive energy and mechanical behaviour. This work is likely to inspire consideration of rare earth-based EQH compounds for application in future spintronic and magneto-electronic devices.

## Abstract 2

Under uniform strain, there are natural physical transitions from spin-filter magnetic semiconductor (MS) to spin-gapless semiconductor (SGS) to half-metal (HM) for rare earth-based equiatomic quaternary Heusler (EQH) compounds with the formula LuCoV*Z*, and from HM → SGS → MS → SGS → HM for EQH compounds with the formula YCoV*Z*.

## 1. Introduction   

In the areas of materials science and solid-state chemistry and physics (Žutić *et al.*, 2004 ▸; Wolf *et al.*, 2001 ▸), neither spintronics nor magneto-electronics can be ignored. In these research fields, the theoretical design of new materials has attracted extensive research interest since it allows *à la carte* design of materials for specific applications (Sato *et al.*, 2010 ▸; Hybertsen & Louie, 1985 ▸). In this connection, much attention has been focused on the Heusler compounds (Graf *et al.*, 2011 ▸; Felser *et al.*, 2015 ▸), because many of them have been found to be half-metal (HM) magnets with high Curie temperatures (Kundu *et al.*, 2017 ▸). In addition to the HM magnets, Heusler-based magnetic semiconductors (MS) (Moodera *et al.*, 2010 ▸) are also of interest for spintronics and magneto-electronics. MSs can be used as spin-filter materials (Miao *et al.*, 2011 ▸) to maximize the efficiency of devices based on magnetic tunnel junctions (MTJs), such as the spin-current diode proposed by Sun & Xie (2015 ▸). Very recently, a new class of materials, spin-gapless semiconductors (SGSs), has been predicted by Wang and co-workers (Wang & Zhang, 2010 ▸; Wang, Cheng, Wang, Rozale *et al.*, 2016 ▸; Wang, 2008 ▸, 2017 ▸). SGSs are a special case of MSs and are also special zero-gap materials. Some of the most important and unique features of SGSs can be summarized as follows: (i) only a tiny amount of energy is required to excite electrons from the valence band to the conduction band; (ii) the excited charge carriers, both electrons and holes, can be 100% spin-polarized simultaneously; (iii) using the Hall effect, fully spin-polarized electrons and holes can be easily separated.

Several investigations (Alijani, Ouardi *et al.*, 2011 ▸; Alijani, Winterlik *et al.*, 2011 ▸; Alijani *et al.*, 2012 ▸; Özdoğan *et al.*, 2013 ▸, 2015 ▸; Bainsla, Suresh *et al.*, 2014 ▸; Bainsla, Mallick *et al.*, 2014 ▸; Galanakis *et al.*, 2013 ▸, 2016 ▸; Galanakis, 2004 ▸; Choudhary *et al.*, 2016 ▸; Gao *et al.*, 2013 ▸, 2015 ▸; Singh *et al.*, 2013 ▸; Benkabou *et al.*, 2015 ▸; Zhang, Liu *et al.*, 2014 ▸; Zhang, Li & Yang, 2014 ▸; Yan *et al.*, 2016 ▸; Wang *et al.*, 2017 ▸; Benkaddour *et al.*, 2016 ▸; Rasool *et al.*, 2016 ▸) have been conducted on HMs and on spin-filter and spin-gapless semiconductors with the equiatomic quaternary Heusler structure (LiMgPdSn/Y structure, space group 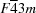, No. 216). Compared with the pseudoternary Heusler HMs, the EQH ones have the advantage of lower power dissipation due to the lower amount of disorder that exists in them (Bainsla & Suresh, 2016 ▸). Also, Heusler-type thin films usually lose their predicted ultra-high spin polarization due to the appearance of disorder. The half-metallic properties of EQH compounds are, however, quite robust against interfering effects (Feng *et al.*, 2015 ▸).

**Figure 1:**
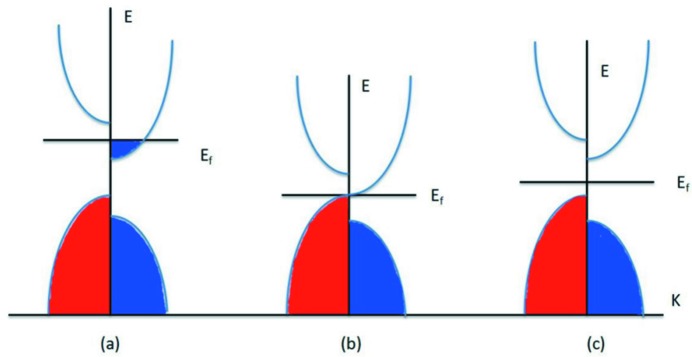
Schematic representations of the density of states (DOS) for (*a*) half-metals, (*b*) spin-gapless semiconductors and (*c*) magnetic semiconductors.

EQH-based HMs exhibit a conducting channel for one spin and a semiconducting/insulating channel for the other, as plotted in Fig. 1 ▸(*a*). To date, many EQH compounds with the formula *XYMZ*, where *X*, *Y* and *M* stand for 3*d* transition metals, have been predicted experimentally and/or theoretically to be novel HMs, such as CoFeMn*Z* (*Z* = Al, Ga, Si, Ge; Alijani, Ouardi *et al.*, 2011 ▸), CoFeCr*Z* (*Z* = Al, Ga, Ge; Gao *et al.*, 2013 ▸) and CoMnCrAl (Mohamedi *et al.*, 2016 ▸). The scope of EQH-based HMs was then extended to compounds including 4*d* transition metals or rare earths, such as ZrCoTi*Z* (*Z* = Al, Ga, Si, Ge; Berri *et al.*, 2014 ▸), ZrFeV*Z* (*Z* = Al, Ga, In; Guo *et al.*, 2016 ▸), YCoTi*Z* (*Z* = Si, Ge; Rasool *et al.*, 2016 ▸) and YCoCr*Z* (*Z* = Si, Ge, Ga, Al; Rasool *et al.*, 2015 ▸). The width of the half-metallic/spin-flipping band gap of these compounds is normally larger than that of EQH compounds that only contain 3*d* transition metals, which is beneficial to the stability of the half-metallicity in practical applications (Zhang, Wang & Cheng, 2017 ▸). Interestingly, our recent work (Zhang, Wang & Cheng, 2017 ▸) demonstrated that the LuCoCrGe EQH compound can become a highly dispersive (near-linear dispersive) zero-gap HM at its strained lattice constant.

SGSs were defined by Wang and co-workers almost a decade ago (Wang & Zhang, 2010 ▸; Wang, Cheng, Wang, Wang & Liu, 2016 ▸; Wang, 2008 ▸, 2017 ▸). According to Wang’s work (Wang, 2008 ▸, 2017 ▸), for SGSs there are four possible band-structure configurations with spin-gapless features. In this work, for clarity, we simplify the four cases into two: (i) one spin direction is gapless, while the other spin direction is semiconducting; (ii) there is a gap between the conduction and valence bands for both the majority and minority electrons, while there is no gap between the majority electrons in the valence band and the minority electrons in the conduction band (see Fig. 1 ▸ *b*). EQH-based SGSs were not paid much attention before Ouardi *et al.* (2013 ▸) confirmed the SGS behaviour of Mn_2_CoAl in bulk-like polycrystalline films. Since then, more and more EQH compounds have been predicted to be SGSs, such as CoMnCrSi (Xu *et al.*, 2013 ▸), ZrCoVIn (Gao *et al.*, 2015 ▸) and CoFeCrGa (Xu *et al.*, 2013 ▸). Note that the CoFeMnSi and CoFeCrGa compounds have been synthesized by Bainsla and co-workers (Bainsla, Mallick *et al.*, 2014 ▸; Bainsla *et al.*, 2015 ▸). We have considered almost all the known EQH SGSs in our review paper (Wang, Cheng, Wang, Wang & Liu, 2016 ▸). Thus far, to the best of our knowledge, rare earth-containing SGSs with the EQH structure have not been investigated by others.

Spin-filter (SF) semiconductors (see Fig. 1 ▸ *c*) are normal MSs which offer a different width of band gap in the two spin-electronic structures and therefore can serve as SFs, finding application as barriers in MTJs. To date, some EQH compounds have been predicted to be SF semiconductors (MSs). In 2013, CoVTiAl and CrVTiAl EQH compounds were identified as MSs by Özdoğan and co-workers (Galanakis *et al.*, 2013 ▸; Galanakis, Özdoğan *et al.*, 2014 ▸). According to their theoretical results, CoVTiAl and CrVTiAl present high Curie temperatures, making these materials suitable for room-temperature spintronics and magneto-electronics applications. Very recently, the polycrystalline compound CrVTiAl has been prepared by Stephen *et al.* (2016 ▸) and Venkateswara *et al.* (2017 ▸). Their experimental results are in good agreement with the previous theoretical studies, namely, CrVTiAl was proved to be a zero-moment SF material with a high Curie temperature above 1000 K. Importantly, MSs with fully compensated ferrimagnetic behaviour have been observed in rare earth-containing EQH compounds, such as CrVY*Z* (*Z* = Si, Ge, Sn) and CrVSc*Z* (*Z* = Si, Ge, Sn) (Özdoğan *et al.*, 2015 ▸).

In the search for new spintronic materials as an ongoing research field, many such compounds, such as new HMs, SGSs and SFSs, are currently attracting enormous attention. Following this research trend, in this work we have used first-principles calculations to study the phase stability and the electronic, magnetic and mechanical properties of four rare earth-containing EQH compounds with the formula *M*CoV*Z*, where *M* = Lu, Y and *Z* = Si, Ge. We demonstrate that all these compounds are SFMs/MSs at their equilibrium lattice constants. Remarkably, a rather rare physical transition and different types of spin-gapless feature can be observed under a uniform strain.

## 2. Computational details   

The electronic, magnetic and mechanical properties of the four compounds were calculated using the pseudopotential method with a plane-wave basis set, as implemented in the *Cambridge Serial Total Energy Package* (*CASTEP*) code. The interactions between the atomic core and the valence electrons were described by the ultrasoft pseudopotential approach (Vanderbilt, 1990 ▸). The exchange and correction between electrons were calculated by the generalized gradient approximation (GGA) (Perdew *et al.*, 1992 ▸, 1996 ▸) in the scheme of Perdew–Burke–Enzerh (PBE). In all cases, a plane-wave basis set (Payne *et al.*, 1992 ▸) cut-off of 450 eV was used. A *k*-point mesh of 15 × 15 × 15 was used in the Brillouin zone integrations for the Heusler structure. These parameters ensured good convergence for the total energy. The convergence tolerance for the calculations was selected as a difference in the total energy within 1 × 10^−6^ eV atom^−1^.

Note that, recently, Bainsla, Kharel and co-workers have successfully prepared samples of CoFeMnSi (Bainsla, Mallick *et al.*, 2014 ▸) and MnCrVAl (Kharel, 2017 ▸) EQH-based SGSs. In this work, to ensure the suitability of *CASTEP* for EQH-based SGSs with relatively subtle band structures, the electronic structures of these two EQH compounds were first calculated using the *CASTEP* code. The theoretical calculation results achieved with the help of GGA-PBE and the experimental results were consistent. Furthermore, in theory, Tas *et al.* (2017 ▸) showed that standard density functional theory (DFT) calculations using GGA-PBE give a fair description of the behaviour of SGSs.

**Figure 2:**
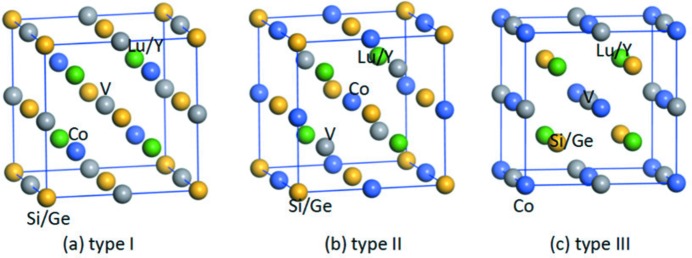
Crystal structures in conventional unit cells for three different types of Wyckoff coordinate for atoms in rare earth-containing EQH compounds with the formula *M*CoV*Z* (*M* = Lu, Y; *Z* = Si, Ge).

Usually, EQH compounds exhibit LiMgPdSn-type (or so-called *Y*-type) structures. As plotted in Fig. 2 ▸ and Table 1 ▸, there are three possible crystal structures with different atomic positions for *M*CoV*Z* EQH compounds. Based on the atom-occupation rule in EQH compounds and similar investigations of EQH systems (Rasool *et al.*, 2016 ▸, 2015 ▸; Zhang, Wang & Cheng, 2017 ▸; Xu *et al.*, 2017 ▸), Y and Lu with fewer valence electrons are inclined to enter the Wyckoff site *D* (0.75, 0.75, 0.75), Co and V tend to occupy the *A* (0, 0, 0) and *C* (0.5, 0.5, 0.5) sites, respectively, and *Z* (*Z* = Si, Ge) atoms tend to be located at the *B* (0.25, 0.25, 0.25) site. For all the rare earth-containing EQH compounds, type III (see Fig. 1 ▸ *c*) is the most stable because it has the lowest energy.

## 3. Results and discussion   

### 3.1. Electronic structure and the Slater–Pauling rule   

In Table 2 ▸, we have given the calculated equilibrium lattice constants, the total and individual atomic magnetic moments, and the number of valence electrons (defined as *Z*_t_) for these four EQH compounds with the type III structure. Obviously, all the EQH compounds in that table obey the well known Slater–Pauling rule (Skaftouros *et al.*, 2013 ▸; Galanakis *et al.*, 2002 ▸; Galanakis, Şaşıoğlu *et al.*, 2014 ▸; Galanakis, 2014 ▸; Shaughnessy *et al.*, 2013 ▸; Birsan & Kuncser, 2016 ▸), namely, the total magnetic moments of these *M*CoV*Z* compounds scale linearly with the number of valence electrons (*M*_t_ = *Z*_t_ − 18).

**Figure 3:**
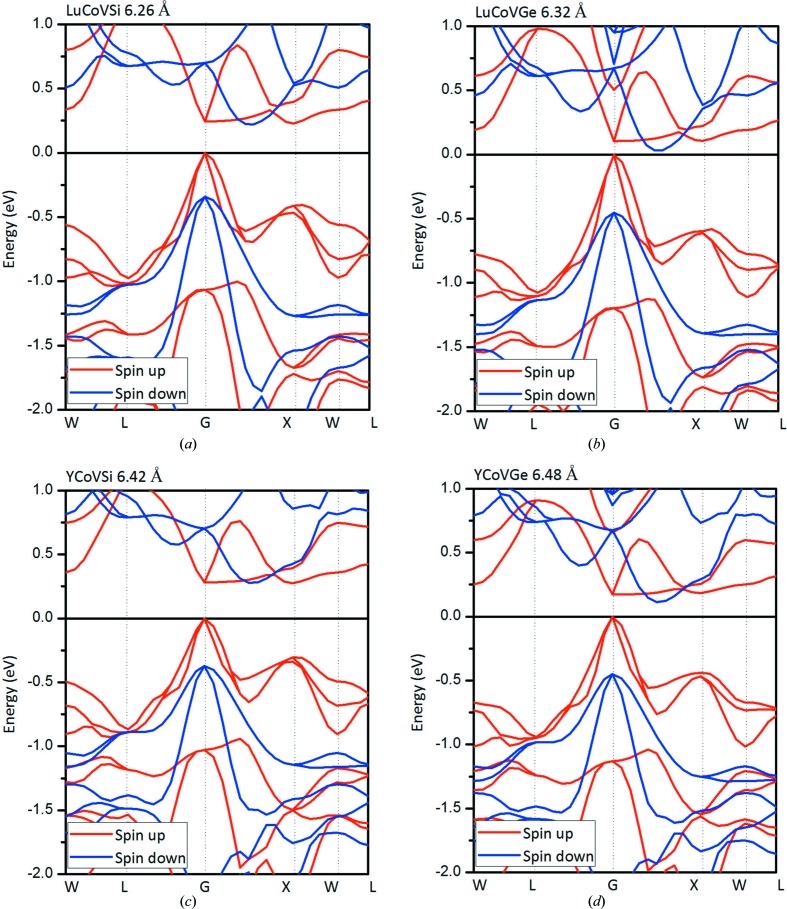
Calculated band structures for the rare earth-containing EQH compounds (*a*) LuCoVSi, (*b*) LuCoVGe, (*c*) YCoVSi and (*d*) YCoVGe at their equilibrium lattice constants.

In Fig. 3 ▸, we present the calculated band structures for these EQH compounds. A clear indirect band gap can be seen in the spin-down channel. However, in the spin-up channel, the band gap is different from that in the spin-down channel: in the spin-up direction, the valence-band maximum touches the Fermi level and a visible energy gap can be observed between the Fermi level and the conduction-band minimum. Therefore, based on the calculated electronic structures and the magnetic moments, these *M*CoV*Z* EQH compounds are MSs/SFMs.

**Figure 4:**
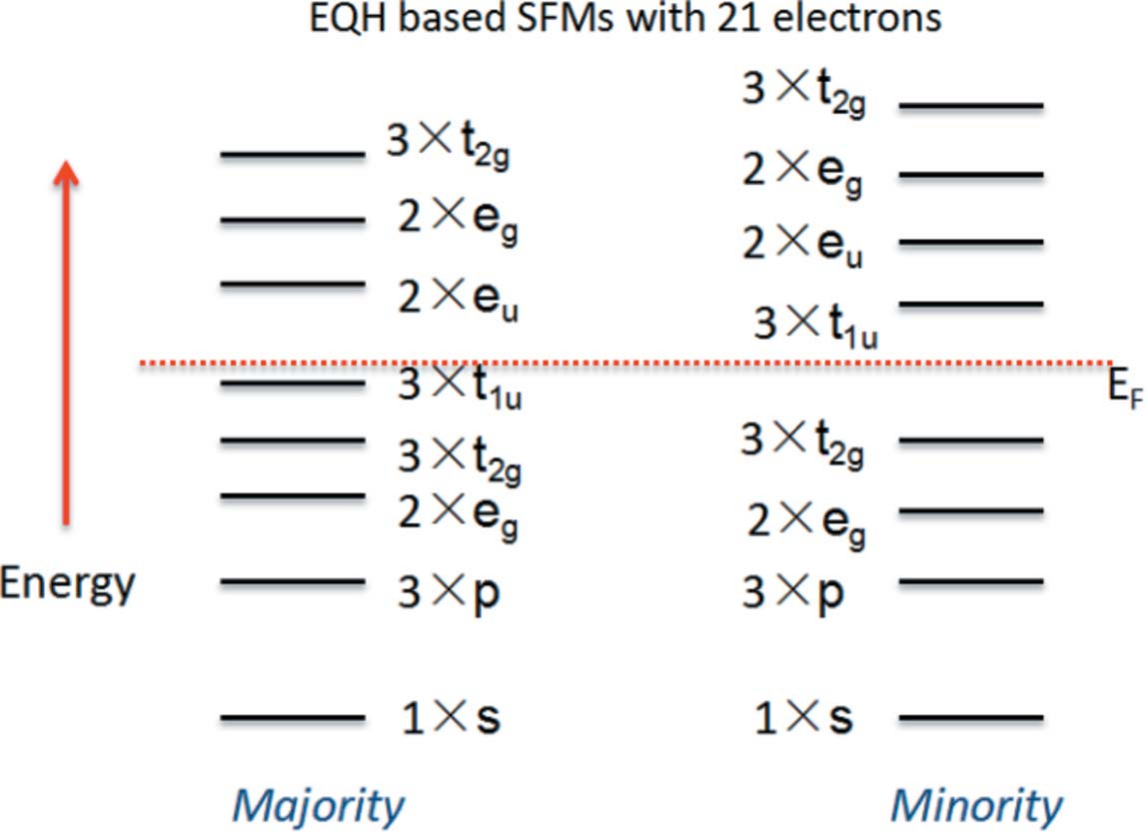
Schematic representation of the band structure for the 21-electron magnetic semiconductors LuCoVSi, LuCoVGe, YCoVSi and YCoVGe in this work.

The difference in the energy gaps between the spin-up and spin-down channels indicates that the mechanisms of these two spin channels are different. To further analyse the origin of the band gaps in the two spin channels, in Fig. 4 ▸ we show a schematic diagram of the energy levels of the spin-up and spin-down band structures for SFMs with 21 valence electrons. From this, we can see that the triple degeneracy *t*_1u_ states are not occupied in the spin-down channel, and therefore a *t*_1u_ (non-bonding)–*t*_2g_ (bonding) energy band gap is formed in the spin-down direction for these *M*CoV*Z* EQH compounds. In the spin-up direction, however, the *t*_1u_ states are fully occupied and the double degeneracy *e*_u_ states are above the Fermi level. That is, an *e*_u_–*t*_1u_ band gap is created in the spin-up channel. Based on the generalized electron-filling rule (Zhang, Xu *et al.*, 2013 ▸; Zhang, Cheng *et al.*, 2017 ▸), for *M*CoV*Z* the total numbers of occupied states are 12 and 9 in the spin-up and spin-down channels, respectively, and therefore there is a total spin magnetic moment of 3 µ_B_.

**Figure 5:**
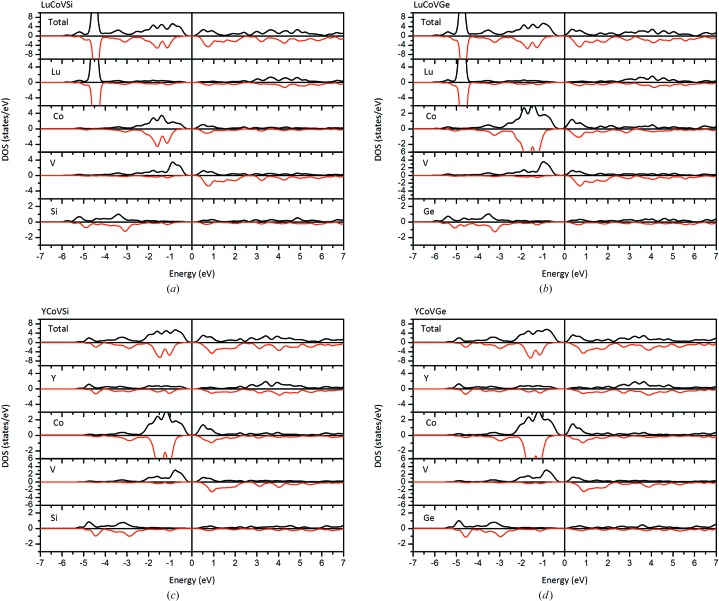
Calculated total and partial DOSs for (*a*) LuCoVSi, (*b*) LuCoVGe, (*c*) YCoVSi and (*d*) YCoVGe EQH compounds.

In Fig. 5 ▸, the total and the partial density of states (TDOS and PDOS, respectively) for the *M*CoV*Z* EQH compounds are presented. The PDOS indicates that the 3*d* atoms Co and V mainly contribute to the TDOS near the Fermi level. Compared with the PDOS of the 3*d* atoms, those of the rare earth and main group atoms are rather lower around the Fermi energy. In Fig. 5 ▸, the V atom shows a strong spin-splitting, with bonding states lying at around −0.8 eV in the spin-up direction and antibonding states lying at around 0.8 eV in the spin-down direction. For the Co atom, the bonding and antibonding states are mainly located in the energy ranges between −2 and −1 eV, and 0 and 1 eV, respectively.

### 3.2. Magnetic properties   

The magnetic behaviour of the *M*CoV*Z* EQH compounds (*M* = Y, Lu; *Z* = Si, Ge) at their equilibrium and strained lattice constants is studied in detail in this section. For the case of the equilibrium lattice constant, all these *M*CoV*Z* compounds show a total spin magnetic moment of 3 µ_B_. As shown in Table 2 ▸, the magnetic moments are mainly localized at the *C* (0.5, 0.5, 0.5) site for V atoms, while the Co atoms at the *A* (0, 0, 0) site carry small magnetic moments aligned parallel to those of the V atoms. Note that the Y and Lu atoms also have induced magnetic moments, which indicates their hybridization with the Co and V atoms.

**Figure 6:**
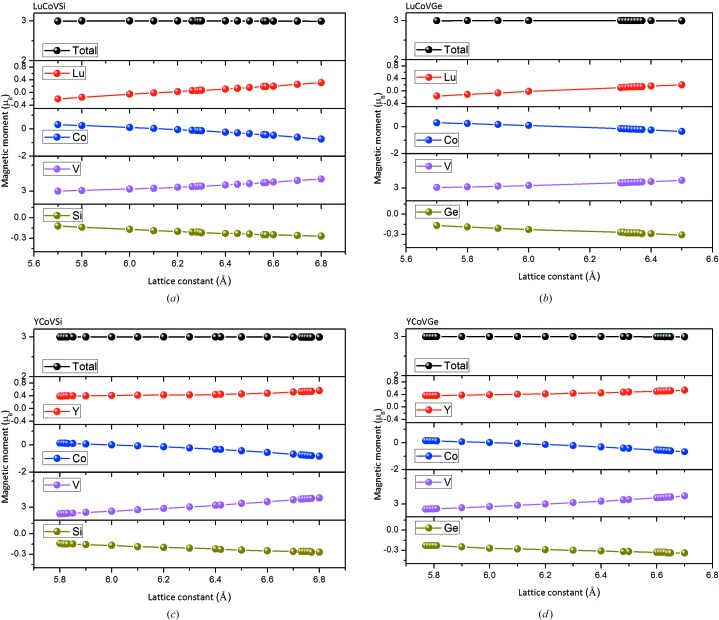
Total and individual atomic magnetic moments as functions of the lattice constants for (*a*) LuCoVSi, (*b*) LuCoVGe, (*c*) YCoVSi and (*d*) YCoVGe EQH compounds.

The total and individual atomic magnetic moments of the *M*CoV*Z* compounds at their strained lattice constants have also been investigated, as shown in Fig. 6 ▸. The findings demonstrate the variation in the partial magnetic moment with respect to contraction and expansion of the lattice constant between 5.70 and 6.80 Å for LuCoVSi, 5.70 and 6.50 Å for LuCoVGe, 5.80 and 6.80 Å for YCoVSi, and 5.77 and 6.70 Å for YCoVGe. For all these EQH compounds, the total magnetic moment is always the fixed integer value of 3 µ_B_ at all the lattice constants mentioned above. The values of the magnetic moments for the Co and *Z* (*Z* = Si, Ge) atoms decrease with increasing lattice constant, whereas for the V and *M* (*M* = Y, Lu) atoms they increase continuously.

### 3.3. Strain-induced diverse transitions of a physical nature   

In this section, we investigate the change in electronic structure under uniform strain. Novel diverse transitions of a physical nature can be obtained by observing the band structures of these *M*CoV*Z* EQH compounds at different lattice constants.

**Figure 7:**
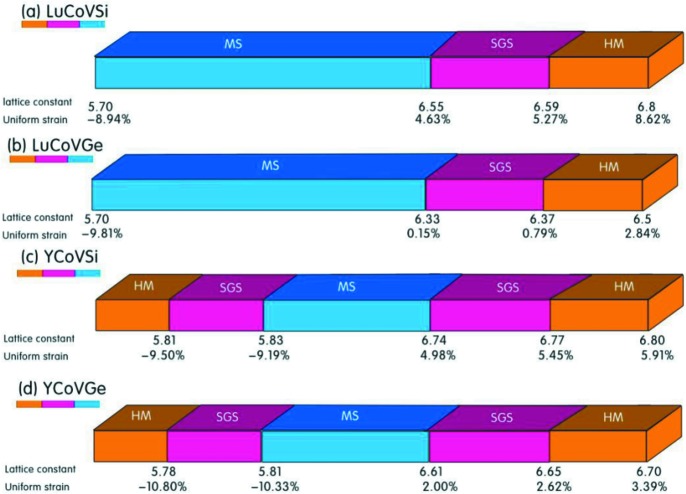
(*a*), (*b*) The MS → SGS → HM transitions under uniform strain for LuCoV*Z* (*Z* = Si, Ge). (*c*), (*d*) The HM → SGS → MS → SGS → HM transitions under uniform strain for YCoV*Z* (*Z* = Si, Ge).

**Figure 8:**
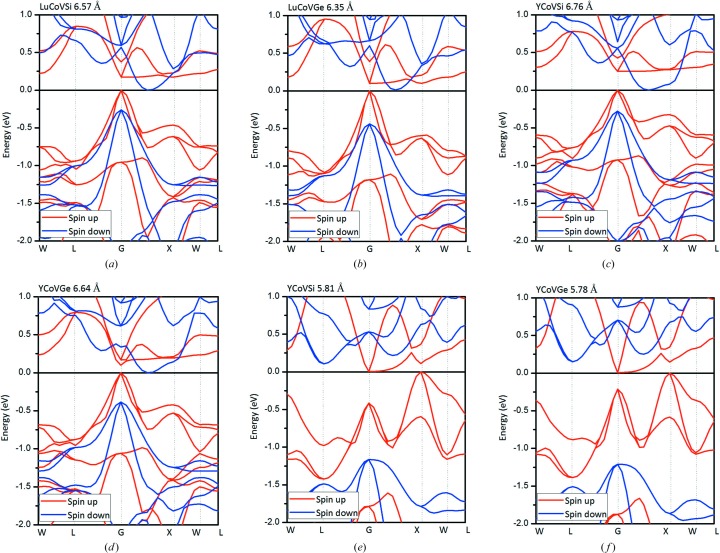
Calculated band structures for the rare earth-containing EQH compounds (*a*) LuCoVSi, (*b*) LuCoVGe, (*c*), (*e*) YCoVSi and (*d*), (*f*) YCoVGe at their strained lattice constants. Different types of spin-gapless behaviour can be clearly observed for these compounds.

**Figure 9:**
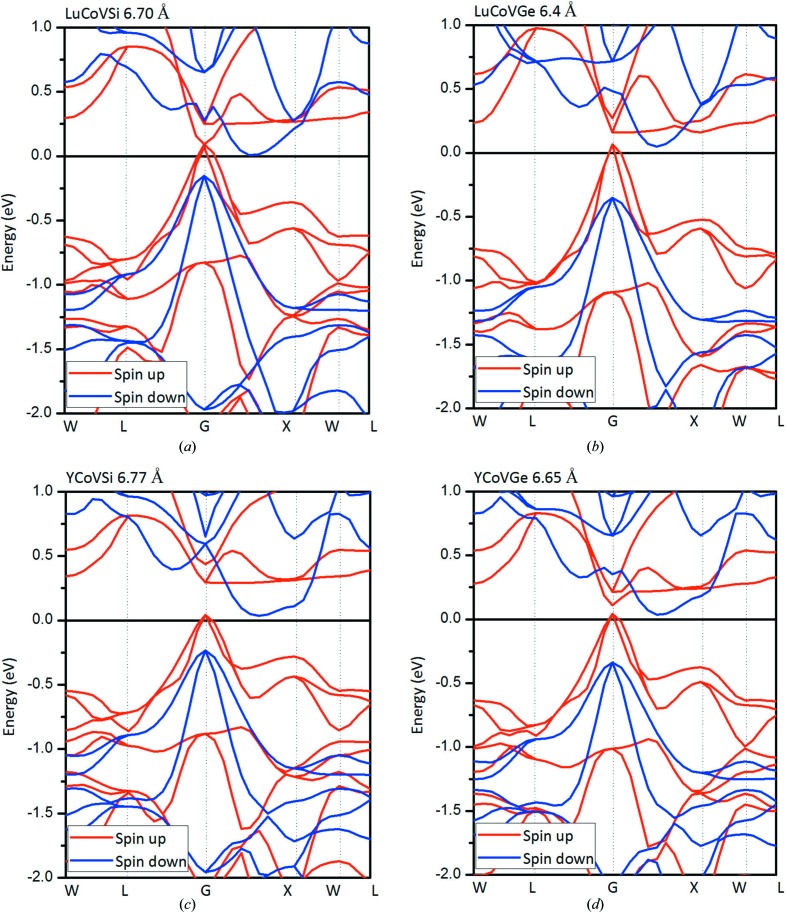
Calculated band structures for the rare earth-containing EQH compounds (*a*) LuCoVSi, (*b*) LuCoVGe, (*c*) YCoVSi and (*d*) YCoVGe at their strained lattice constants. The half-metallic properties of these compounds are obvious.

As shown in Figs. 7 ▸(*a*) and 7 ▸(*b*), we observe that there is a novel transition from an MS/SFM to an SGS and an HM for the LuCoVSi and LuCoVGe EQH compounds. The corresponding band structures are also summarized in Figs. 8 ▸ and 9 ▸. In detail, as the lattice constant is expanded in the ranges 6.55–6.59 Å for LuCoVSi, and 6.33–6.37 Å for LuCoVGe (see the examples of LuCoVSi 6.57 Å and LuCoVGe 6.35 Å in Fig. 8 ▸), the conduction bands in the spin-down channel move down, and a zero gap between the majority electrons in the valence band and the minority electrons in the conduction band occurs, *i.e.* the LuCoV*Z* (*Z* = Si, Ge) compounds become SGSs (type II). As the lattice constants increase further (6.59–6.80 Å for LuCoVSi, and 6.37–6.50 Å for LuCoVGe), the valence bands at the *G*-point in the spin-up channel move up and also cross the Fermi level (see the examples of LuCoVSi and LuCoVGe at the lattice constants of 6.70 and 6.40 Å, respectively, in Fig. 9 ▸), and therefore the LuCoV*Z* (*Z* = Si, Ge) compounds become HMs.

For the case of the YCoV*Z* (*Z* = Si, Ge) EQH compounds, the transitions have novel physics and form MS → SGS (type II) → HM under uniform strain in the ranges −9.19–5.91% and −10.33–3.39%, respectively, as can also be observed in Figs. 7 ▸(*c*), 7 ▸(*d*) and 8 ▸ (see the examples of YCoVSi 6.76 Å and YCoVGe 6.64 Å) and Fig. 9 ▸ (see the examples of YCoVSi 6.77 Å and YCoVGe 6.65 Å).

For a deeper understanding, by comparing the band structures in Figs. 8 ▸ and 9 ▸, the principal reasons for the changing physics of the transitions can be summarized as follows (Wang, Cheng, Wang, Rozale *et al.*, 2016 ▸; Wang, Cheng, Wang & Liu, 2016 ▸): (i) changes in the spin splitting; (ii) changes in the valence- and conduction-band energies; and (iii) changes in the degree of dispersion of the valence and conduction bands.

### 3.4. Different types of strain-induced spin-gapless features   

Remarkably, with decreasing lattice constants (5.81–5.83 Å for YCoVSi, 5.78–5.81 Å for YCoVGe), the valence bands at the *X*-point (the conduction bands at the *G*-point) in the spin-up channel move up (down) and touch the Fermi level, and a zero gap can be found in the spin-up channel (see the examples of YCoVSi and YCoVGe at the lattice constants of 5.81 and 5.78 Å, respectively, in Fig. 8 ▸), and therefore both compounds become SGSs (type i).

Note that this is the first time that different types of spin-gapless feature have been observed in an EQH compound. As shown in Fig. 8 ▸, the band structures of SGS YCoVSi and YCoVGe compounds can be different and quite complicated. In particular, the appearance of a zero-width gap at the Fermi level is a rare phenomenon (Jamer *et al.*, 2013 ▸), and therefore both compounds with tunable spin-gapless features deserve more attention.

### 3.5. Mechanical properties   

In order to discover the mechanical stability and understand the mechanical properties of these *M*CoV*Z* EQH compounds (*M* = Lu, Y; *Z* = Si, Ge), their elastic nature has been revealed by calculating the elastic constants and their related behaviour. For cubic crystals, only three independent elastic constants, *C*_11_, *C*_12_ and *C*_44_, are taken into account, and these independent elastic constants are used to calculate all of the elastic modulus matrix using the following equations (Cherid *et al.*, 2017 ▸): 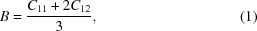


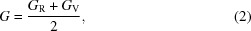


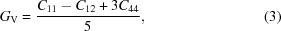


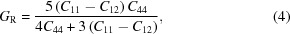


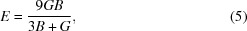


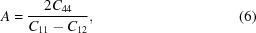
where *G* stands for the shear modulus, *B* is the bulk modulus, *G*_V_ is the Voigt shear modulus, *G*_R_ is the Reuss shear modulus, *E* is the Young’s modulus and *A* is the anisotropy factor.

First, the mechanical stability of these *M*CoV*Z* EQH compounds was tested based on the generalized elastic stability criteria given by Born & Huang (1954 ▸): 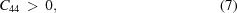


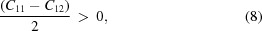


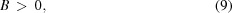


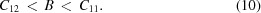
As shown in Table 3 ▸, we observed that these mechanical stability criteria were satisfied for *M*CoV*Z* (*M* = Y, Lu; *Z* = Si, Ge). Therefore, these EQH compounds are mechanically stable.

Second, the Young’s modulus of a material is the usual property used to characterize stiffness. Normally, the higher the value of *E*, the stiffer the material. Thus, the relative stiffness order for our current EQH compounds is YCoVGe > LuCoVSi > YCoVSi > LuCoVGe. Furthermore, the values of *B*/*G* are equal to 2.10 for LuCoVSi, 1.89 for LuCoVGe, 2.05 for YCoVSi and 1.85 for YCoVGe, reflecting that all four EQH compounds in this work are ductile based on Pugh’s criteria (Pugh, 1954 ▸). The relative ductility order is LuCoVSi > YCoVSi > LuCoVGe > YCoVGe.

Finally, the anisotropy factor (*A*) was calculated to predict the anisotropic or isotropic behaviour of each compound. As shown in Table 3 ▸, we observe that the values of *A* are not equal to unity, reflecting the fact that these rare earth-containing EQH compounds are anisotropic.

### 3.6. Formation and cohesive energies   

In this section, we discuss the phase stability of the rare earth-containing *M*CoV*Z* EQH compounds based on their formation and cohesive energies, with the help of GGA+PBE. These types of calculation can help us to test whether these EQH compounds could be synthesized experimentally. We should point out that there have been similar investigations of the phase stability of many ternary and quaternary Heusler compounds (Wei *et al.*, 2012 ▸; Ahmadian & Salary, 2014 ▸; Kervan & Kervan, 2013 ▸; Zhang, Liu *et al.*, 2013 ▸; Fan *et al.*, 2009 ▸; Chen *et al.*, 2015 ▸, 2011 ▸; Birsan, 2014 ▸).

First, we calculated the cohesive energies (*E*_c_) of *M*CoV*Z* compounds 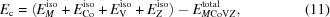
where 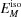, 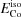, 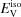 and 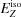 are the isolated atomic energies of the rare earth atoms *M* (*M* = Y, Lu), Co, V and the main group element *Z* (*Z* = Si, Ge), respectively, and 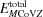 is the total energy of *M*CoV*Z* per formula unit. The calculated values of the cohesive energy are listed in Table 3 ▸. The values are very large and greater than 20 eV for these EQH compounds, which indicates that *M*CoV*Z* crystals are expected to be stable.

Second, the formation energies (*E*_f_) of *M*CoV*Z* compounds were also calculated: 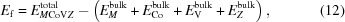
where 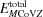 is the total energy of *M*CoV*Z* per formula unit, and 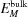, 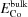, 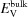 and 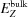 are the total energies per atom of each element in the bulk for the body-centred cubic *M* and Co, hexagonal close-packed V, diamond Si and cubic close-packed Ge, respectively. The negative formation energies (see Table 3 ▸) also indicate the structural stability of these rare earth-containing EQH compounds, and thus these compounds may be synthesized experimentally by conventional equilibrium methods such as arc-melting.

In fact, we should point out that a large number of rare earth-containing ternary Heusler compounds based on the half-Heusler structure have already been prepared successfully (Hou, Wang, Xu, Zhang, Wei *et al.*, 2015 ▸; Hou, Wang, Xu, Zhang, Liu *et al.*, 2015 ▸; Wang *et al.*, 2012 ▸; Zhang *et al.*, 2016 ▸; Mun *et al.*, 2016 ▸). The rare earth-containing EQH compounds presented here have both novel electronic states and negative formation energies at the same time, and experimental realisation of them is feasible and imminent.

## 4. Summary   

Based on the first-principles method, the electronic, magnetic and mechanical properties and the origin of the band gaps of the newly designed rare earth-based *M*CoV*Z* EQH compounds (*M* = Lu, Y; *Z* = Si, Ge) have been investigated in this work. The calculated results indicate that these compounds are MSs at their equilibrium lattice constants. At strained lattice constants, the LuCoV*Z* and YCoV*Z* compounds undergo interesting physics, changing from MS → SGS (type II) → HM and HM → SGS (type I) → MS → SGS (type II) → HM, respectively, which means that the electronic and magnetic structures could be extensively tuned by external temperature or pressure. Importantly, tunable spin-gapless features were also found in YCoV*Z* compounds subjected to strain engineering. Because of their diverse electronic and magnetic properties, the present work suggests that all these rare earth-containing *M*CoV*Z* EQH compounds could be useful in spintronic applications.

The phase stability of these EQH compounds was also examined from the points of view of their formation and cohesive energies and their mechanical behaviour. Our results show that these rare earth-containing EQH compounds are very likely to be feasible for experimental synthesis by conventional equilibrium methods such as arc-melting.

## Figures and Tables

**Table 1:** Atomic positions of the crystal structure at different crystal sites for the three types of *M*CoV*Z* (*M* = Lu, Y; *Z* = Si, Ge) compounds

Type	Lu/Y	Co	V	Si/Ge
Type 1	*D* (0.75, 0.75, 0.75)	*B* (0.25, 0.25, 0.25)	*C* (0.5, 0.5, 0.5)	*A* (0, 0, 0)
Type 2	*D* (0.75, 0.75, 0.75)	*C* (0.5, 0.5, 0.5)	*B* (0.25, 0.25, 0.25)	*A* (0, 0, 0)
Type 3	*D* (0.75, 0.75, 0.75)	*A* (0, 0, 0)	*C* (0.5, 0.5, 0.5)	*B* (0.25, 0.25, 0.25)

**Table 2:** Total and individual atomic magnetic moments (μ_B_), calculated equilibrium lattice constant, number of valence electrons, and possible Slater–Pauling (S-P) rule for the EQH compounds *M*CoV*Z* (*M* = Lu, Y; *Z* = Si, Ge) with type III structure

Compound	Total	Lu/Y	Co	V	*Z*	*a* (Å)	*Z*_t_	S-P rule
LuCoVSi	3.00	0.06	−0.10	3.26	−0.21	6.26	21	*M*_t_ = *Z*_t_ − 18
LuCoVGe	3.00	0.12	−0.16	3.31	−0.28	6.32	21	*M*_t_ = *Z*_t_ − 18
YCoVSi	3.00	0.45	−0.34	3.12	−0.23	6.42	21	*M*_t_ = *Z*_t_ − 18
YCoVGe	3.00	0.47	−0.39	3.24	−0.32	6.48	21	*M*_t_ = *Z*_t_ − 18

**Table 3:** Calculated elastic constants *C*_*ij*_, bulk modulus *B*, shear modulus *G*, Young’s modulus *E* (GPa), anisotropy factor *A*, Pugh’s ratio *B*/*G*, and formation and cohesive energies (eV) for the EQH compounds *M*CoV*Z* (*M* = Lu, Y; *Z* = Si, Ge) with type III structure

EQH compound	*C*_11_	*C*_12_	*C*_44_	*B*	*G*	*E*	*A*	*B*/*G*	Formation energy	Cohesive energy
LuCoVSi	174.8	60.7	41.1	98.8	46.9	121.5	0.72	2.10	−0.83	22.05
LuCoVGe	142.3	47.8	38.6	79.3	41.8	106.8	0.81	1.89	−0.47	22.54
YCoVSi	158.3	56.5	40.0	90.4	44.0	113.7	0.78	2.05	−1.07	23.05
YCoVGe	167.3	67.7	57.7	100.9	54.4	138.3	1.15	1.85	−0.32	23.65
